# *Asparagus cochinchinensis* Extract Alleviates Metal Ion-Induced Gut Injury in* Drosophila*: An In Silico Analysis of Potential Active Constituents

**DOI:** 10.1155/2016/7603746

**Published:** 2016-03-31

**Authors:** Weiyu Zhang, Li Hua Jin

**Affiliations:** College of Life Science, Northeast Forestry University, Harbin 150040, China

## Abstract

Metal ions and sulfate are components of atmospheric pollutants that have diverse ways of entering the human body. We used* Drosophila* as a model to investigate the effect of* Asparagus cochinchinensis* (*A. cochinchinensis*) extracts on the gut and characterized gut homeostasis following the ingestion of metal ions (copper, zinc, and aluminum). In this study, we found that the aqueous* A. cochinchinensis* extract increased the survival rate, decreased epithelial cell death, and attenuated metal ion-induced gut morphological changes in flies following chronic exposure to metal ions. In addition, we screened out, by network pharmacology, six natural products (NPs) that could serve as putative active components of* A. cochinchinensis* that prevented gut injury. Altogether, the results of our study provide evidence that* A. cochinchinensis* might be an effective phytomedicine for the treatment of metal ion-induced gut injury.

## 1. Introduction

In recent years, atmospheric pollution has become a rapidly growing international trend. Atmospheric pollution not only is harmful to the Earth's climate, agriculture, and industry but also does immeasurable damage to humans. Atmospheric pollution can cause respiratory system damage, physiological abnormalities, neurological abnormalities, and digestive disorders [1, 2]. Metal ions and sulfate are components of atmospheric pollution stemming primarily from fuel combustion and large-scale industrial and mining enterprises, as well as from other man-made pollutants such as exhaust gas. Metal ions enter the human body in diverse ways, including inhalation, swallowing, and skin contact. Due to their nondegradation characteristics, metal ions accumulate in the body where they are converted into more toxic metal compounds by combining with organic matter. This triggers a series of damaging effects that result in physiological dysfunctions [3]. Previous studies have demonstrated that a dose of cadmium induces intestinal epithelial cell injury in the* Drosophila* midgut [4]. The intestinal epithelial is an important protective barrier between the internal and external environment. Mechanisms of immunity and tissue regeneration must be tightly regulated in order to maintain intestinal homeostasis [5, 6]. Dysregulation of inflammatory responses and tissue regeneration can lead to inflammatory bowel diseases and colorectal cancer in mammals [7]. In recent years, increasingly more people are plagued by intestinal inflammation, and prolonged inflammation and tissue damage can lead to intestinal carcinogenesis and tumor formation.

*Asparagus cochinchinensis* (*A. cochinchinensis*), referred to as Tiandong in China, is the root of* A. cochinchinensis* (Lour.) Merr. (Liliaceae) that is distributed among many provinces of China.* A. cochinchinensis* (AC) has been used in traditional Chinese medicine (TCM) for over 2,000 years. Its flavor is sweet, bitter, and cold. The channel tropism is the lungs, kidney, stomach, and large intestine meridian.* A. cochinchinensis* has often been used for the treatment of fever, cough, throat pain, swelling, constipation, and diabetes. The dried root has antibacterial, antipyretic, diuretic, expectorant, stomachic, nervous stimulant, and tonic properties [8]. Modern research has also demonstrated that* A. cochinchinensis* has antitumor activity, especially in lung cancer [9, 10]. However, the protective effect on intestinal injury and the active components of* A. cochinchinensis* affecting gut immunity remain poorly understood.

*Drosophila* is a well studied and highly tractable genetic model organism. Many basic biological and physiological properties are conserved between* Drosophila* and mammals, and nearly 75% of human disease-related genes have a functional homolog in the fly [11]. Thus, the* Drosophila* is widely used in basic and applied researches on a broad spectrum of human diseases including infectious diseases [12], cancers [13], neurodegenerative diseases [14], and metabolic diseases [15].* Drosophila* and human intestine have similar anatomy and physiological function [16, 17], and they have also similarities in cell and composition and underlying signaling pathways that maintain intestinal homeostasis [18].

Chinese medicinal herbs exert their therapeutic actions through the synergistic effects of multiple compounds, targets, and channels. However, it has been difficult to isolate the effective components of these products and to identify specific therapeutic targets for treating disease. Network pharmacology has been used as an integrated approach to systematically investigate and explain the underlying molecular mechanisms of Chinese medicinal herbs. Using the Computerized Virtual Screening Technique to explore potential targets may help facilitate these investigations, while reducing manpower and material resources.

*Drosophila* has emerged as a potential whole animal model for drug screening [19, 20]. Through a large number of survival rate assays, we identified* A. cochinchinensis* as having good bioactivity against chemical reagents-induced stress in* Drosophila* (data was not shown). In this study, we revealed that the aqueous extract of* A. cochinchinensis* exerts a protective effect on gut injury in* Drosophila* induced by the chronic exposure to metal ions. In addition, we computationally identified the putative active ingredients of* A. cochinchinensis* using network pharmacology. Network analysis revealed six constituents of* A. cochinchinensis* that could potentially mediate its protective effects on gut injury. These results provide new insight into the pharmacological basis of the antigut injury activity of* A. cochinchinensis* and will provide impetus for preclinical drug discovery based on this medicinal plant.

## 2. Materials and Methods

### 2.1. *Drosophila* Fly Stocks

*w* ^*1118*^ flies were obtained from the Bloomington* Drosophila* Stock Center. Fly stocks were maintained on a 12 h light/12 h dark cycle at 25°C and 60% humidity.

### 2.2. *A. cochinchinensis* Extraction

*A. cochinchinensis* was identified by Professor Xiuhua Wang at the Herbarium of the College of Life Sciences, Northeast Forestry University, and purchased from Shiyitang Pharmacy of Harbin, China. The method of aqueous extraction of* A. cochinchinensis* has been previously described [21]. Total aqueous-derived extract was consolidated and concentrated to a final concentration of 20% (w/v).

### 2.3. *Drosophila* Food

Standard cornmeal-yeast medium used for the control group consisted of 5.6 g/L agar, 16.8 g/L yeast, 71.6 g/L polenta, 9.8 g/L soybean flour, and 60 g/L sucrose. Standard medium with 10% (w/v) aqueous* A. cochinchinensis* extract was used for the experimental group.

### 2.4. Survival Experiments

Procedures for the survival and feeding experiments were performed as previously described [21] with the difference that adult flies which aged 3–5 days (15 males and 15 females) were starved for 2 h without food before being transferred to a vial containing chemical compounds in 5% (w/v) sucrose solution serving as the control group for all experiments. The chemical compounds included cupric sulfate (7 mM, Sigma), zinc sulfate (7 mM, Sigma), and aluminum potassium disulfate dodecahydrate (20 mM, Sigma). The experimental group consists of flies fed with sucrose solutions incorporating the chemical compound with added AC extract at 10% (w/w). Fresh filter papers and solutions were provided every day, and dead flies were enumerated and evaluated daily.

### 2.5. 7-Amino-actinomycin D Staining and Imaging

7-Amino-actinomycin D (7-AAD) staining and imaging were performed as previously described [21, 22].

### 2.6. Intestinal Morphological Analysis

Female flies were used for intestinal morphological studies due to their larger size.

Due to the bigger size of female flies, we used them for intestinal morphological analysis experiment. The guts of 3–5-day-old female flies that had orally ingested metal ions in 5% (w/v) sucrose with or without AC extract for 96 h were dissected at room temperature in phosphate buffered saline (PBS) and immediately observed under an Axioskop 2 plus microscope (Zeiss).

### 2.7. Targets Predictions of Natural Products (NPs) and Network Construction

The information pertaining to natural products (NPs) was obtained from the Universal Natural Products Database (UNPD) according to the scientific names of the herbs [23]. The information for approved drugs and drug targets was obtained from DrugBank [24]. We used the PharmMapper server to predict potential drug targets [25]. The network was constructed by Cytoscape 3.2.1 [26].

### 2.8. Statistics

For all experiments, data are representative of three independent experiments. Statistical analyses, including Student's* t*-test, the one-way ANOVA, and log-rank (Mantel-Cox) test, were performed using GraphPad Prism 5.0 software (^*∗*^ *P* < 0.05; ^*∗∗*^ *P* < 0.01; ^*∗∗∗*^ *P* < 0.001; ns: no significant difference).

## 3. Results

### 3.1. *A. cochinchinensis* Increased Survival following the Ingestion of Metal Ions

As water decoction is the traditional formulation used in Chinese clinical medicine, we used aqueous extraction in this study. To assess the effect of* A. cochinchinensis* (AC) extract on the development of* Drosophila* larvae, we orally administered different concentrations of AC extract to flies from egg until the adult stage. We found that AC extract demonstrated no cytotoxicity at doses of 10% (w/v) (data not shown); therefore, we used this concentration for the experiments described in this paper.

**Figure 1:**
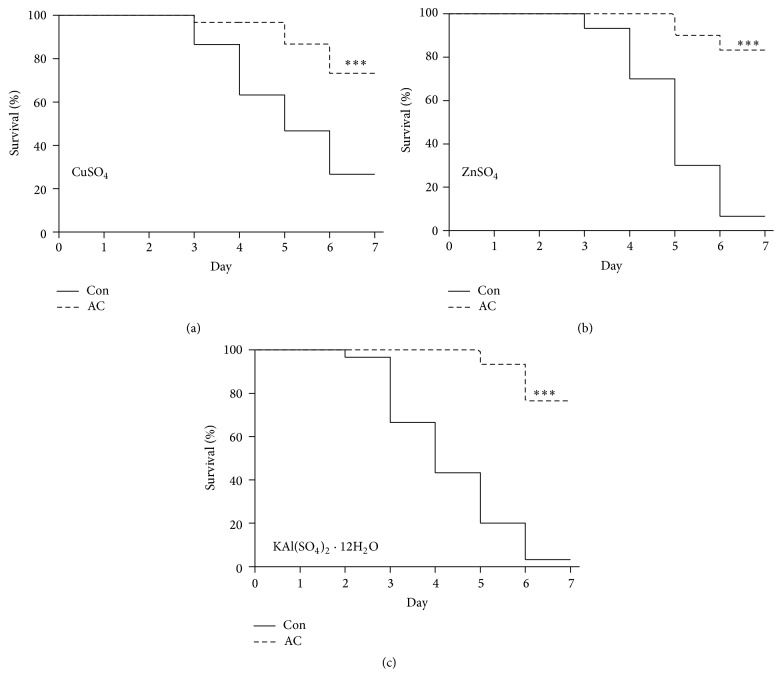
*A. cochinchinensis* improves the survival rate of flies that have ingested metal ions. (a) The survival rates of AC extract and control flies fed with 7 mM CuSO_4_. (b) The survival rates of AC extract and control flies fed with 7 mM ZnSO_4_. (c) The survival rates of AC extract and control flies fed with 20 mM KAl(SO_4_)_2_·12H_2_O. Three replicates were used for the determination of survival rates. *P* value was calculated by the log-rank test. ^*∗∗∗*^ *P* < 0.001 was considered statistically significant.

In order to investigate the effect of AC extract on the* Drosophila* gut following metal ion ingestion, we first examined whether AC extract affected survival. We observed that the survival rate of flies fed with AC extract was significantly greater compared with the control group following six days of metal ion ingestion. The survival rates associated with Cu ingestion were 74.4% for the AC extract group and 25% for the control group (Figure 1(a)). Six days following ingestion of Zn and Al, the survival rates were 83.3% and 77.8% in the AC extract group and 5.2% and 2.4% in the control group, respectively (Figures 1(b) and 1(c)). These results indicate that AC extract can increase survival in flies that have ingested metal ions. Based on this observation, we hypothesized that AC extract might have a protective effect on metal ion-induced gut injury in* Drosophila*.

### 3.2. *A. cochinchinensis* Protects the Gut from Metal Ion-Induced Epithelial Cell Death in* Drosophila*

**Figure 2:**
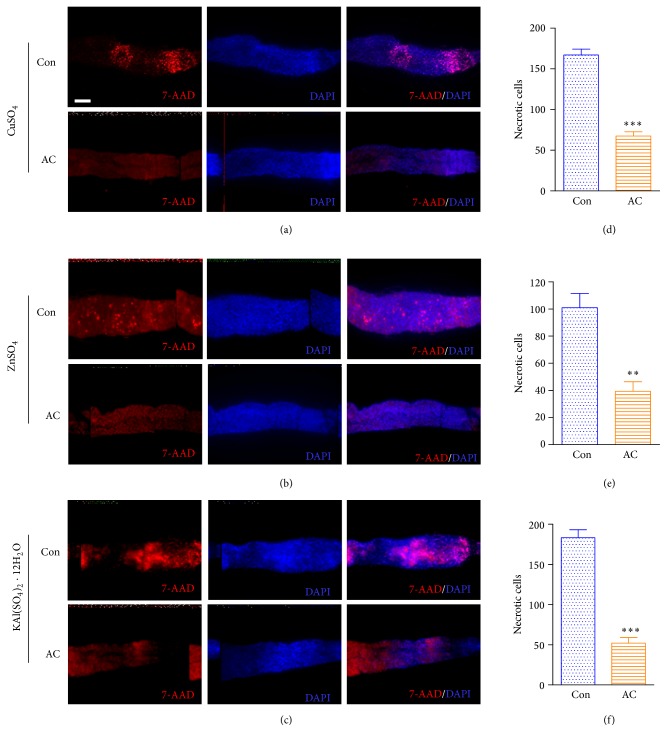
*A. cochinchinensis* protects gut epithelial cells from metal ion-induced cell death. (a) The effect of AC extract on Cu-induced epithelial cell death. (b) The effect of AC extract on Zn-induced epithelial cell death. (c) The effect of AC extract on Al-induced epithelial cell death. ((d)–(f)) The comparison of gut necrotic cell quantity between control and AC group. The number of necrotic cells was quantified using ImageJ software (10–15 guts were examined to quantify necrotic cells for each group). *P* value was calculated by Student's *t*-test. ^*∗∗∗*^ *P* < 0.001; ^*∗∗*^ *P* < 0.01. Con: control, flies fed with metal ions without AC for 96 h; AC: flies fed with metal ions with* A. cochinchinensis* (AC) for 96 h. 7-AAD: 7-amino-actinomycin D, dead cells (red signal); DAPI: 4′,6-diamidino-2-phenylindole, nucleus (blue signal); 7-AAD/DAPI (red and blue signal). Scale bar: 100 *μ*m.

We further examined our hypothesis that AC has protective effects on metal ion-induced gut epithelial cell injury by evaluating gut epithelial cell death. After 4 days of ingesting various metal ions with or without AC extract, we found that Cu and Al feeding was associated with significantly more cell death compared with Zn feeding (Figure 2). Furthermore, only very few dead cells were detected in the AC extract group compared with the control group (Figure 2, red signal). These results demonstrate that AC extract can maintain host homeostasis by protecting gut epithelial cells from metal ion-induced damage.

### 3.3. *A. cochinchinensis* Protects the Gut from Metal Ions-Induced Morphological Changes in* Drosophila*

**Figure 3:**
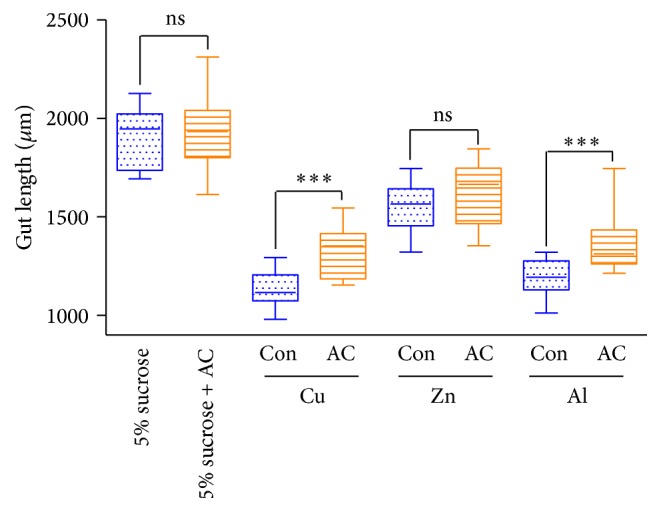
*A. cochinchinensis* prevents metal ion-induced gut atrophy. Con: control, flies fed with metal ion without AC for 96 h; AC:* A. cochinchinensis*, flies fed with metal ion with AC for 96 h. All experiments were independently performed three times. ^*∗∗∗*^ *P* < 0.001; ns: no significant difference.

A large number of reactive oxygen species (ROS) were rapidly produced after feeding some toxic compounds [21, 22]. However, excessive ROS can damage the host intestinal epithelial cells. Study has shown that cadmium could change membrane permeability through inhibition of superoxide dismutase activity and result in necrotic organelles [27]. A previous study has shown that an increase in gut epithelial cell death associated with morphological changes in* Drosophila* [28]; therefore, we examined the gut morphology of flies that had been fed with metal ions. Four days after induction, we observed that the length of the adult gut from the control groups was significantly shorter than that of the group fed with 5% (w/v) sucrose (Figure 3). In contrast, the length of AC groups was alleviated compared with control groups which were fed with Cu and Al. However, there was no significant difference between the control group and the AC group following Zn feeding (Figure 3). These observations suggest that AC extract prevents metal ion-induced morphological changes in the adult fly gut, and, altogether, the results from our study demonstrate that AC extract has a protective effect on metal ion-induced gut injury in* Drosophila*.

### 3.4. The Prediction of Potential Targets of* A. cochinchinensis* Natural Products

Based on our observations, we hypothesized that AC has potential implications for the discovery of new intestinal anti-inflammatory drugs. To test this hypothesis, we obtained 29 natural products (NPs) derived from AC from the UNPD. Next, we screened out 19 therapeutic proteins targeted by FDA-approved intestinal anti-inflammatory agents from DrugBank. Finally, using PharmMapper server, we found that 19 of the 29 NPs were predicted to bind to 3 of the 19 proteins targeted by FDA-approved intestinal anti-inflammatory agents (Supplementary Table S1 in Supplementary Material available online at http://dx.doi.org/10.1155/2016/7603746). These three targets were corticosteroid 11-beta-dehydrogenase isozyme 1 (11-DH), glucocorticoid receptor (GR), and peroxisome proliferator-activated receptor gamma (PPAR*γ*). PharmMapper server is a web server for potential drug target identification using pharmacophore mapping approach [25, 29, 30].

**Figure 4:**
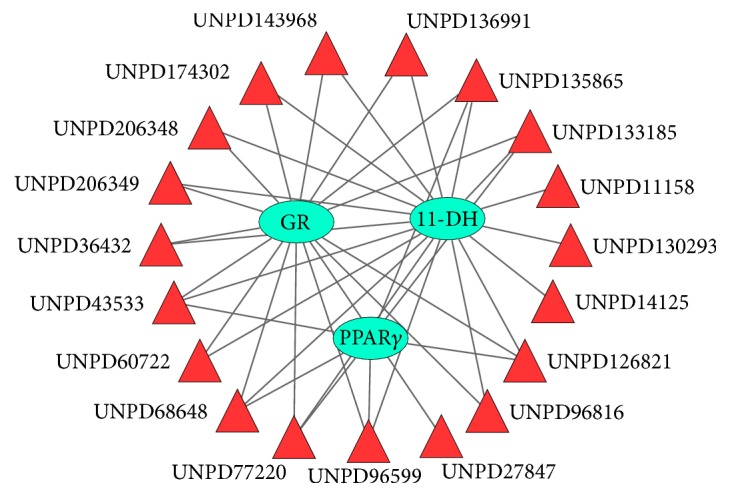
The NP-target network. The target protein was expressed by a node, and edges represented the relationship between NPs and the target protein. 11-DH: corticosteroid 11-beta-dehydrogenase isozyme 1; GR: glucocorticoid receptor; PPAR-gamma: peroxisome proliferator-activated receptor gamma. UNPD ID: Universal Natural Products Database identification.

Cytoscape software is a popular bioinformatics package for biological network visualization and data integration [26, 31]. According to the docking results, we constructed a NP and target network using the Cytoscape 3.2.1 network analysis software. The target protein was expressed by a node, and the edges represented the relationship between NPs and targets (Figure 4). Using this network, we found that 18 NPs targeted 11-DH, 16 NPs targeted GR, and 7 NPs targeted PPAR*γ*. This result indicates that AC might serve as a useful new medicine for treating intestinal injury.

### 3.5. The Putative Components of* A. cochinchinensis* That Mediate Protection from Intestinal Injury

Oral administration, a simple, low-cost option that does not directly damage mucous membranes, has become the most commonly used mode of drug administration. Our results demonstrated that 7* A. cochinchinensis* NPs are predicted to bind three proteins targeted by FDA-approved intestinal anti-inflammatory agents simultaneously (Figure 4). We narrowed down the list of 7 candidates to 6 NPs by applying Lipinski's rule of five (Table 1) and considered these to be the putative components that mediate the protective effect of AC on intestinal injury.

### 3.6. The Drug-Likeness of the 6 NPs

**Figure 5:**
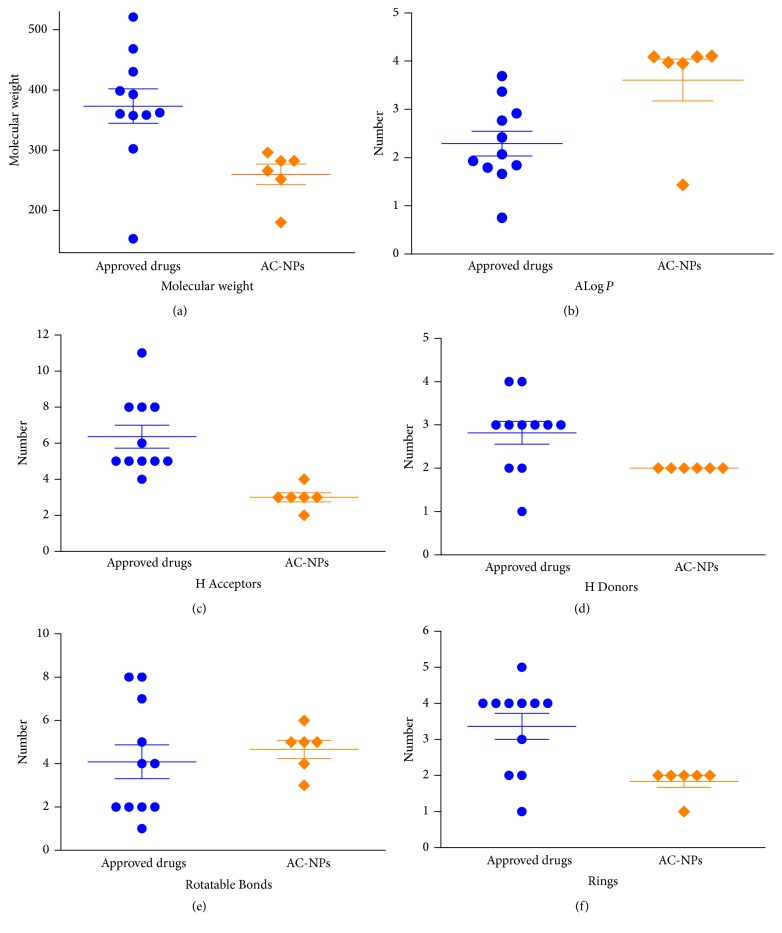
Distribution of the six molecular descriptors of AC-NPs and FDA-approved drugs. H Acceptors: number of hydrogen bond receptors; H Donors: number of hydrogen bond donors; Rotatable Bonds: number of rotatable bonds; Rings: number of rings. Approved drugs: FDA-approved drugs; AC-NPs:* A. cochinchinensis* natural products.

Molecular descriptors have been extensively used in cheminformatics research and the pharmaceutical industry for molecular clustering [32]. In order to explore the drug-likeness of the 6 NPs screened out from* A. cochinchinensis* (AC-NPs), we gathered information on 11 FDA-approved intestinal anti-inflammatory agents from DrugBank (Supplementary Table S2) and calculated the following six descriptors: molecular weight, ALog *P*, number of hydrogen bond receptors (H Acceptors), number of hydrogen bond donors (H Donors), number of rotatable bonds (Rotatable Bonds), and number of rings (Rings) (Supplementary Tables S2 and S3). Using this information, we drew scatter plots to facilitate analysis of drug-likeness. As shown in Figure 5, the mean values of the descriptors of AC-NPs tended to be smaller than those of FDA-approved drugs. Nevertheless, ALog *P* of AC-NPs was larger than that of approved drugs. The mean value of ALog *P* was 3.6 ± 1.1 for AC-NPs and 2.3 ± 0.8 for the FDA-approved drugs. The numbers of H Acceptors and H Donors of approved drugs were primarily 5 and 3, but they were 3 and 2 for AC-NPs. The mean number of rotatable bonds for AC-NPs was slightly larger than those of the approved drugs, and this amounted to a significant difference. Most of the approved drugs had approximately 2 rotatable bonds, while the AC-NPs had approximately 5. These results indicate that the 6 AC-NPs have good drug-likeness and could potentially be used as intestinal anti-inflammatory drugs.

## 4. Discussion

*A. cochinchinensis* is primarily used in obstetrics and gynecology, otolaryngology, and ophthalmology. Its use in the clinical setting has revealed its remarkable antiaging, antitumor, and antiproliferative effects [33–35]. Previous studies have shown that AC ethanol extract might have therapeutic potential in immune-related cutaneous diseases [36]. However, the effect of AC aqueous extract on gut injury has not been previously characterized. In this study, AC significantly ameliorated epithelial cell damage in adult flies following chronic metal ion feeding (Figure 1). Due to the high level of conservation between* Drosophila* and mammalian intestinal properties [18], our results provide a theoretical basis for exploring the potential use of AC for the clinical treatment of inflammatory bowel disease.

Reverse docking of a small molecule compound (natural products, lead compounds, and chemicals) to a probe is an approach used to predict potential drug targets. It is an important tool for drug research and is indispensable for driving the modernization of new drug discovery. In this study, using the PharmMapper server, we observed AC-NP docking with targets that participate in several signaling pathways, including those associated with cancer, lipid metabolism, neurodegenerative diseases, the primary immunodeficiency pathway, and nitrogen metabolism. Moreover, some NPs demonstrated docking with intestinal anti-inflammatory targets. These observations support the hypothesis that AC exerts protective effects on metal ion-induced gut injury in* Drosophila*.

PPAR*γ* belongs to a subfamily of the nuclear hormone receptor superfamily of ligand-inducible transcription factors [37]. PPAR*γ* regulates genes related to lipid metabolism, as well as genes associated with immunity and inflammation [38–41]. In our study, we observed UNPD126821, UNPD133185, UNPD135865, UNPD43533, UNPD68648, UNPD77220, and UNPD96599 docking with PPAR*γ* (Figure 4). This result further supported the hypothesis that AC has a protective effect on metal ion-induced gut injury in* Drosophila*. Moreover, this result provided a potential mechanism by which AC mediates its protective effects on gut injury. Altogether, our results suggest that AC extracts may employ similar pharmacological mechanisms as western medicines to prevent gut injury.

Nyasol was isolated from* A. cochinchinensis* many years ago, but there are few reports on its pharmacological properties [42]. Nyasol has demonstrated anti-inflammatory effects in LPS-activated BV-2 microglial cells [43]. In our study, we found that UNPD43533 (Nyasol) targeted 11-DH, GR, and PPAR*γ* (Figure 4). This result demonstrates that AC has the potential to be used as an intestinal anti-inflammatory drug. The molecular descriptors of Nyasol were consistent with Lipinski's rules of five (Supplementary Table S3), indicating that Nyasol has good drug-likeness and is not likely to be associated with absorption problems. Therefore, Nyasol has potential implications in intestinal anti-inflammatory drug discovery. In addition, we found that Nyasol docked with estradiol 17-beta-dehydrogenase 1 (HSD17B1), the insulin receptor, and other molecules. HSD17B1 is known to be involved in lipid transport and metabolism, and the insulin receptor is a key regulator of the insulin signaling pathway. Therefore, we predict that Nyasol may also have potential as an antidiabetic agent; however, this hypothesis requires further experimental verification.

## 5. Conclusion

Our results demonstrate that* A. cochinchinensis* aqueous extract exerts a protective effect on metal ion-induced gut injury in* Drosophila*. In addition, we screened out six constituents of* A. cochinchinensis* that could potentially mediate this effect. In addition, these NPs are associated with good drug-likeness. Further studies will be required to delineate the pharmacological properties associated with each of the putative active components of* A. cochinchinensis* and to determine the mechanism by which* A. cochinchinensis* mediates its protective effects in metal ion-induced gut injury. Taken together, our findings provide a basis to support the potential use of* A. cochinchinensis* for intestinal inflammation. Moreover, our studies provide helpful information and new insights into support of the application of TCM-derived natural products to drug discovery and development.

## Supplementary Material

29 natural products (NPs) of *A. cochinchinensis* obtained from Universal Natural Products Database (UNPD). 11 FDA-approved intestinal anti-inflammatory drugs and 19 targets collected from DrugBank. 19 of the 29 NPs were predicted to target with 3 of the 19 proteins by PharmMapper server. 6 NPs accorded with Lipinski's rule of five.
The message was seen in Supplementary Material.

## Figures and Tables

**Table 1:** The putative components of AC that mediate intestinal injury protection.

UNPD ID	Chemical name	CAS number	CID
UNPD133185	Coniferyl alcohol	32811-40-8|458-35-5	1549095
UNPD43533	Nyasol	230292-85-0	6438674
UNPD77220	Asparenydiol	166762-98-7	10084256
UNPD135865	3′-Hydroxy-4′-methoxy-4′-dehydroxynyasol	N/A	21575014
UNPD68648	3′′-Methoxyasparenydiol	N/A	N/A
UNPD96599	3′′-Methoxynyasol	N/A	25218067
