# Supplementary material for: Asparagus cochinchinensis Extract Alleviates Metal Ion-Induced Gut Injury in Drosophila: An In Silico Analysis of Potential Active Constituents
Source: Evid Based Complement Alternat Med. 2016 Mar 31;2016:7603746. doi: 10.1155/2016/7603746 (PMC4830720; doi:10.1155/2016/7603746)
Supplement: Supplementary file 1 — 29 natural products (NPs) of A. cochinchinensis obtained from Universal Natural Products Database (UNPD). 11 FDA-approved intestinal anti-inflammatory drugs and 19 targets collected from DrugBank. 19 of the 29 NPs were predicted to target with 3 of the 19 proteins by PharmMapper server. 6 NPs accorded with Lipinski's rule of five. The message was seen in Supplementary Material. [file 7603746.f1.pdf]

Table S1 The list of NPs screened from PharmMapper server.

No.	UNPD ID	Chemical_Name	CAS_NO.	PubChem CID
1	**UNPD133185**	Coniferyl alcohol	32811-40-8|458-35-5	1549095
2	**UNPD27847**	Pseudoprotodioscinbeta	102115-79-7|117557-44-5	N/A
3	**UNPD36432**	Pseudoprotodioscin	102115-79-7	N/A
4	**UNPD43533**	Nyasol	230292-85-0	6438674
5	**UNPD60722**	(25S)-5beta-spirostan-3beta-yl-O-[O-alpha-L-rhamnosyl (1->4)]-beta-D-glucopyranoside	58881-26-8	N/A
6	**UNPD77220**	Asparenydiol	166762-98-7	10084256
7	**UNPD11158**	Asparacoside	N/A	21575006
8	**UNPD126821**	Pseudoprotodioscin	N/A	N/A
9	**UNPD130293**	Asp-VI	N/A	N/A
10	**UNPD135865**	3'-Hydroxy-4'-methoxy-4'-dehydroxynyasol	N/A	21575014
11	**UNPD136991**	Asparacosin A	N/A	11385250
12	**UNPD14125**	Asparacoside	N/A	21575006
13	**UNPD143968**	1,3-bis(4-hydroxyphenyl)pent-4-en-1-one	N/A	N/A
14	**UNPD174302**	Asparacosin B	N/A	21575013
15	**UNPD206348**	Aspacochioside C	N/A	N/A
16	**UNPD206349**	Aspacochioside D	N/A	N/A
17	**UNPD68648**	3''-Methoxyasparenydiol	N/A	N/A
18	**UNPD96599**	3''-Methoxynyasol	N/A	25218067
19	**UNPD96816**	Asp-V	N/A	N/A

Table S2 The list of FDA approved intestinal anti-inflammatory agents.

No.	Drug name	Drug ID	CAS NO.	Molecular Formula	Molecular Weight	ALogP	Num H Acceptors	Num H Donors	Num Rotatable Bonds	Num Rings
**1**	**Sulfasalazine**	DB00795	599-79-1	$C_{18}H_{14}N_4O_5S$	398.393	2.92	8	3	5	3
**2**	**Balsalazide**	DB01014	80573-04-2	$C_{17}H_{15}N_3O_6$	357.3175	3.37	8	4	7	2
**3**	**Olsalazine**	DB01250	15722-48-2	$C_{14}H_{10}N_2O_6$	302.239	2.77	8	4	4	2
**4**	**Mesalazine**	DB00244	89-57-6	$C_7H_7NO_3$	153.1354	0.75	4	3	1	1
**5**	**Cromoglicic Acid**	DB01003	16110-51-3	$C_{23}H_{16}O_{11}$	468.3665	1.84	11	3	8	4
**6**	**Prednisone**	DB00635	1953-3-2	$C_{21}H_{26}O_5$	358.4281	2.07	5	2	2	4
**7**	**Beclomethasone**	DB00394	5534-9-8	$C_{28}H_{37}ClO_7$	521.042	3.69	5	1	8	4
**8**	**Budesonide**	DB01222	51333-22-3	$C_{25}H_{34}O_6$	430.5339	2.42	6	2	4	5
**9**	**Prednisolone**	DB00860	50-24-8	$C_{21}H_{28}O_5$	360.444	1.66	5	3	2	4
**10**	**Betamethasone**	DB00443	378-44-9	$C_{22}H_{29}FO_5$	392.4611	1.93	5	3	2	4
**11**	**Hydrocortisone**	DB00741	50-23-7	$C_{21}H_{30}O_5$	362.4599	1.79	5	3	2	4

Table S3 The list of NPs accorded with Lipinski's rule of five.

No.	UNPD ID	Chemical Name	Molecular Weight	ALogP	Num H Acceptors	Num H Donors	Num Rotatable Bonds	Num Rings
**1**	**UNPD133185**	Coniferyl alcohol	180.2	1.434	3	2	3	1
**2**	**UNPD135865**	3'-Hydroxy-4'-methoxy-4'-	282.334	4.089	3	2	5	2
**3**	**UNPD43533**	Nyasol	252.308	4.105	2	2	4	2
**4**	**UNPD68648**	3''-Methoxyasparenydiol	296.317	3.954	4	2	6	2
**5**	**UNPD77220**	Asparenydiol	266.291	3.971	3	2	5	2
**6**	**UNPD96599**	3''-Methoxynyasol	282.334	4.089	3	2	5	2
